# Uncertainty Quantification of Central Canal Stenosis Deep Learning Classifier From Lumbar Sagittal T2‐Weighted MRI

**DOI:** 10.1002/jsp2.70184

**Published:** 2026-06-08

**Authors:** Adriana Brenzikofer, Maria Monzon, Fabio Galbusera, Zina‐Mary Manjaly, Andrea Cina, Catherine R. Jutzeler

**Affiliations:** ^1^ Department of Health Sciences and Technology ETH Zurich Zurich Switzerland; ^2^ Swiss Institute of Bioinformatics (SIB) Lausanne Switzerland; ^3^ Department of Teaching, Research and Development Schulthess Clinic Zurich Switzerland; ^4^ Department of Neurology Schulthess Clinic Zurich Switzerland

**Keywords:** central canal stenosis, convolutional neural networks, lumbar MRI, *uncertainty estimation*

## Abstract

**Background:**

Accurate assessment of the severity of central canal stenosis (CCS) on lumbar spine MRI is critical for clinical decision‐making. We evaluated deep learning models for automated CCS grading on sagittal T2‐weighted MRI, focusing on uncertainty quantification to improve clinical reliability.

**Methods:**

Using a retrospective cohort from the LumbarDISC dataset (1974 patients), we compared multiple deep learning architectures for three‐level CCS classification (normal/mild, moderate, severe). To assess model confidence, Monte Carlo (MC) dropout and Test Time Augmentation (TTA) techniques were applied to quantify prediction uncertainty.

**Results:**

The fine‐tuned Spine Grading Network (SGN) achieved a balanced accuracy of 79.4% and a macro F1 score of 68.8%, with per‐class accuracies of 71.3% for moderate and 78.5% for severe stenosis. MC dropout revealed an increase in uncertainty predominantly in moderate and severe cases, while TTA uncertainty was higher for mild stenosis.

**Conclusion:**

DL‐based CCS grading demonstrates potential to assist radiologists by providing rapid, standardized evaluations. Incorporating uncertainty quantification offers a safeguard to flag ambiguous cases, thus supporting clinical trust and facilitating safer integration of AI tools into the interpretation of spine MRI.

## 1 Introduction

Low back pain (LBP) remains the leading cause of years lived with disability worldwide, often leading to diminished mobility and independence [1]. One significant aspect of LBP is lumbar spinal stenosis (LSS). This is characterized by narrowing of the spinal canal, which can compress the spinal cord and/or nerve roots, often leading to pain, numbness and disability [2]. The diagnosis of LSS is based on clinical history, physical examination, and Magnetic Resonance Imaging (MRI) of the lumbar spine [3]. However, interpreting MRI scans is complex and time consuming, involving multiple slices and intervertebral levels. This can lead to inconsistent classification among radiologists [4] and delayed diagnoses. In addition, clinical presentation and radiological presentation do not necessarily correlate.

Deep learning (DL) models have emerged as a promising solution to address these gaps, with the potential to assist clinicians by automating the detection and grading of LSS, as well as other pathological findings observed in radiological imaging. Several DL models have been developed to detect and classify LSS [5, 6, 7, 8, 9, 10]. Among these, SpineNetV2 [7, 11] is considered the most advanced model. It was developed to detect vertebral levels and intervertebral discs (IVD) and grade degenerative findings, including central canal stenosis (CCS), a form of LSS characterized by compression or inflammation of the spinal cord. SpineNetV2 has also been externally validated in different clinical datasets [12, 13, 14, 15]. However, a major barrier to the clinical adoption of DL models is to ensure their trustworthiness; without quantifying prediction uncertainty, these models remain difficult to trust or implement in clinical practice [16].

To address this challenge, it is essential to understand that uncertainty in DL models can be broadly classified into epistemic and aleatoric types [17]. Epistemic uncertainty, often referred to as model uncertainty, arises from limitations in the model's knowledge that are typically due to insufficient diverse training data or inadequate model architecture [18]. Monte Carlo (MC) dropout [19] is widely used to estimate epistemic uncertainty. Aleatoric uncertainty is the inherent randomness or irreducible noise in the data that cannot be resolved by adding more training samples [17]. This type of uncertainty in LSS MRI can arise from motion artifacts, anatomical variability, and ambiguous tissue boundaries with overlapping signal intensities that obscure the spinal canal. Test‐Time Augmentation (TTA) is a common method to quantify this data‐inherent uncertainty [20]. Although uncertainty estimation methods such as MC dropout and TTA are used in medical imaging [21, 22], their effectiveness to improve model reliability and help clinical evaluations is underexplored.

**FIGURE 1:**
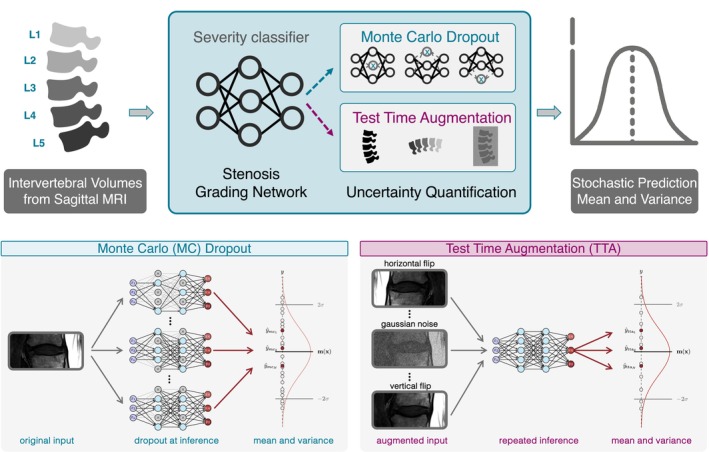
Overview of the uncertainty quantification pipeline for central canal stenosis grading. The pipeline extracts intervertebral volumes from sagittal T2‐weighted lumbar spine MRIs, from intervertebral levels L1‐L2 to L5‐S1. A fine‐tuned Spine Grading Network (SGN), adapted from the pre‐trained SpineNetV2 ResNet‐34 architecture incorporating dropout predicts severity of the stenosis (normal/mild, moderate, severe). To quantify uncertainty, two methods are employed to generate stochastic predictions: Test‐Time Augmentation (TTA) to estimate data‐inherent aleatoric uncertainty and Monte Carlo (MC) Dropout to estimate model‐based epistemic uncertainty. Multiple forward passes through the network yield a distribution of predictions for each input volume.

In this study, we developed a robust DL model for classifying CCS severity [23] from sagittal MRI T2‐weighted scans, emphasizing the critical role of uncertainty quantification in clinical settings. Although calibration analysis assessed the alignment between predicted probabilities and real‐world outcomes, calibrated probabilities alone do not fully capture predictive uncertainty [24, 25]. To better quantify uncertainty, we used MC dropout and TTA, which capture predictive variability beyond standard softmax scores. The main objectives were to (1) compare different DL architectures to classify CCS severity, (2) quantify prediction uncertainty using MC dropout and TTA, and (3) investigate the use of majority voting in the MC and TTA outputs to improve model performance. An overview of the full methodology and uncertainty estimation pipeline is provided in Figure 1. This approach aims to address clinical needs by providing automated CCS classification accompanied by confidence measures, thereby supporting better decision making through explicit awareness of uncertainty.

## 2 Materials and Methods

### 2.1 Dataset Preprocessing

The data set used in this study consisted of sagittal T2‐weighted MRI scans of the lumbar spine from the LumbarDISC dataset [26], comprising 1974 patients across eight international medical centers. Radiologists evaluated and annotated five disc levels (L1‐L2 to L5‐S1) per patient using a three‐tier classification system: normal/mild, moderate, and severe stenosis. Following SpineNetV2 pipeline [7], pre‐processing included slice‐wise normalization to the range [0, 1], with the median intensity of adjacent vertebral bodies that comprise the IVD level set to 0.5. Intervertebral volumes were extracted using the SpineNetV2 detection module, which automatically localizes each disc level and centers the 9‐slice sagittal stack on the mid‐sagittal plane, consistently capturing the central spinal canal across scanners and imaging protocols. The preprocessed volumes of the final input had dimensions of 9 × 112 × 224 voxels (num slices × height × width).

### 2.2 CCS Classification Model

SpineNetV2 was originally trained on the Genodisc dataset [11]. It combines vertebral detection with different classification subnetworks to predict specific degenerative conditions, including the four severity grades of CCS: normal, mild, moderate, and severe based on the Lee classification system [23]. We adapted the SpineNetV2 stenosis classification subnetwork to predict CCS severity according to the three‐tier RSNA challenge system: normal/mild, moderate and severe stenosis. Specifically, the final layer of the ResNet34‐based SpineNetV2 Grading Network (SGN) was adapted to predict three classes instead of four. Adapting a pre‐trained network to a new, related task and retraining only part of the network is known as fine‐tuning. In addition, we applied dropout regularization [27] to various layers of the adapted SGN. Dropout regularization is a technique used during neural network training in which certain neurons are randomly deactivated to encourage the model to generalize better and avoid over‐reliance on specific features. In our model, dropout was applied between layer 4 and the average pooling layer, as well as before the final layer. This step was necessary to enable MC dropout, which allows the model to estimate predictive uncertainty. To benchmark the performance of the fine‐tuned SGN, we also evaluated three alternative DL architectures: DenseNet [28], ResNet [29], and EfficientNet [30], all implemented within the Medical Open Network for Artificial Intelligence (MONAI) framework [31]. The final hyperparameter configurations for all evaluated architectures are detailed in Table S1.

For the development of DL models, we divided our MRI data into 70% for model training, 15% for validation during development, and 15% for final testing, ensuring that a single patient did not appear in multiple splits, avoiding data leakage and ensuring that the proportions of the three stenosis severity grades were similar in all sets. We evaluated the model on the test set using balanced accuracy to address class imbalance, and macro F1 score to capture the trade‐off between precision and recall across all stenosis severity grades. Additionally, we computed the confusion matrix and, for each class, calculated precision (the proportion of cases predicted as severe that are truly severe) and per‐class accuracy (the proportion of true severe cases correctly identified). To assess regional performance differences, we also conducted class‐specific evaluations at each level of the intervertebral disc (IVD).

### 2.3 Uncertainty Estimation

We performed an analysis of the calibration of the model's output probabilities using the Expected Calibration Error (ECE) per class [32]. An ECE of zero means that the probabilities are perfectly calibrated and can be used as a measure of uncertainty. However, since the output probabilities of the DL model are often miscalibrated [24], to enhance the clinical applicability of our top performing CCS classification model, we implemented two uncertainty estimation methods: MC dropout [19] and TTA [20].

MC dropout offers a quantitative evaluation of the epistemic uncertainty of the DL model by maintaining random neuron deactivation during prediction [27]. This technique extends conventional dropout regularization beyond training by maintaining stochastic neuron deactivation during inference, generating multiple predictions from identical input data. The variance of the resulting predictions directly correlates with the uncertainty of the model: higher variance signifies greater uncertainty of the model and, consequently, reduced confidence in any individual prediction, highlighting cases requiring radiologist review. We implemented 25 dropout iterations, as it was observed that, beyond 20 iterations, the predictive uncertainty exhibited minimal variation [33].

To quantify aleatoric uncertainty, which arises from inherent data variability, we implemented TTA [20]. In TTA, augmentations serve as controlled input perturbations rather than realistic clinical simulations. Therefore, anatomical realism is not a prerequisite for valid aleatoric uncertainty estimation. In each TTA iteration, we applied transformations such as horizontal flipping, rotation, scaling, or the addition of Gaussian noise to the image (see Figure S2). Running the model on these modified scans generates a prediction distribution that quantifies uncertainty through the predictive mean and variance. The variance of the multiple runs measures how much these predictions vary from one another, serving as a direct quantification of aleatoric uncertainty.

For both MC dropout and TTA, we computed the mean and standard deviation of predicted probabilities associated with the ground‐truth class across all iterations. Higher variance indicates greater uncertainty. As a complementary uncertainty measure, we also computed predictive entropy from the mean class probability distribution across repeated stochastic predictions. We conducted a comparative visualization of uncertainty estimations between MC and TTA methods. The predictions were categorized according to the severity of the stenosis (normal/mild, moderate, severe) and further filtered by true labels to assess the alignment between the predicted results and actual diagnoses for each method.

This approach ensures consistency and interpretability, as it allows one to directly quantify uncertainty in the model's prediction with respect to the correct diagnosis. The low and high uncertainty cases were identified by analyzing the distribution of standard deviations of predicted probabilities for the true class, computed separately for each method and each class. Images with standard deviations above the 75th percentile were marked as highly uncertain, while those below the 25th percentile were considered low uncertainty. Confidence, in contrast, was evaluated based on the mean predicted probability for the true class: higher values indicate more confident predictions. It is important to distinguish between confidence and uncertainty. Confidence reflects the model's belief in a particular prediction, while uncertainty captures how stable and consistent that belief is across multiple stochastic iterations.

#### 2.3.1 Uncertainty‐Driven Consensus for Reliable CCS Grading

In addition to using TTA and MC for uncertainty quantification, we combined both methods with majority voting, as TTA with majority voting has shown significant performance improvements in medical image analysis [34]. Building on this, TTA and MC dropout were further leveraged by aggregating the predictions through majority voting. For each method, we systematically combined the predictions in iterations (25 for MC dropout, 9 for TTA) to derive more reliable stenosis classifications. The majority voting procedure summed class‐specific predictions across iterations, with the class receiving the highest vote count designated as the final prediction.

## 3 Results

### 3.1 Data Analysis

This study retrospectively used T2‐weighted sagittal magnetic resonance imaging of the lumbar spine of 1974 patients from the LumbarDISC dataset [26]. The resolution of the scans ranges from 0.27 × 0.27 × 3 mm to 1.18 × 1.18 × 6 mm, with a mean number of slices 17.01 ± 2.8.

**FIGURE 2:**
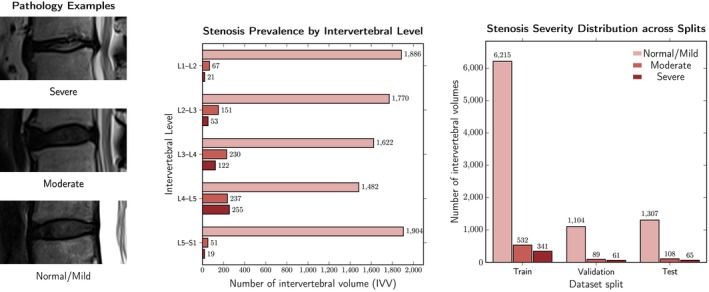
(a) Examples of central canal stenosis severity. (b) Distribution of stenosis severity for each of intervertebral level. (c) Distribution of stenosis severity of intervertebral disc (IVD) labels across train, validation, and test splits and individual patients per split.

After applying the SpineNetV2 detection module, 9830 volumes of intervertebral disc (IVD) were extracted for the training of the CCS network. The anatomical distribution revealed severe stenosis mainly in L4‐L5 and moderate in L3‐L4. Figure 2 shows sample sagittal slices of IVD volumes at level L3‐L4 for the three classes of severity of the stenosis. The data set exhibited a substantial class imbalance, with 88% (*n* = 8,630) of IVD volumes classified as normal/mild stenosis, while moderate and severe stenosis represented only 7% (*n* = 732) and 5% (*n* = 468) of the dataset, respectively.

### 3.2 CCS Classification Model Performance

In the comprehensive evaluation of the five deep learning architectures for lumbar spinal stenosis classification, the fine‐tuned SGN demonstrated superior performance, in terms of macro F1 (68.8%) and balanced accuracy (79.4%). Consequently, this model was selected as the best performing architecture for further analysis and uncertainty estimation procedures. The remaining deep learning architectures exhibited comparatively lower performance in key evaluation metrics. DenseNet achieved the second highest performance with a balanced accuracy of 69.0% and a macro F1 score of 68.7%, closely followed by ResNet (balanced accuracy: 65.4%, macro F1: 65.6%) and EfficientNet (balanced accuracy: 65.6%, macro F1: 66.3%). In particular, the original SpineNetV2 architecture demonstrated the poorest overall performance, with the lowest macro F1 score (59.6%) and balanced accuracy (55.4%) among all evaluated models.

Class‐specific analysis (Table 1) showed different performance patterns in severity categories of stenosis. Detailed confusion matrices for all five architectures are provided in Figure S1. The original SpineNetV2 model had variable performance, with an accuracy of 98.2% for normal/mild stenosis, but only 20.4% and 47.7% for moderate and severe stenosis. The fine‐tuned SGN showed the highest accuracy: 88.4% for normal/mild, 71.3% for moderate and 78.5% for severe stenosis.

**TABLE 1:** Performance evaluation comparison for lumbar spinal central canal stenosis (CCS) classification.

Metric (%)	Fine‐tuned SGN	SpineNetV2	DenseNet	ResNet	EfficientNet
Accuracy					
Normal/mild	88.4	98.2	96.3	96.1	96.4
Moderate	71.3	20.4	53.7	55.6	48.1
Severe	78.5	47.7	56.9	44.6	52.3
Balanced accuracy	79.4	55.4	69.0	65.4	65.6
F1 score					
Normal/mild	93.5	95.6	96.8	96.5	96.6
Moderate	45.8	27.0	48.7	49.0	44.8
Severe	67.1	56.4	60.7	51.3	57.6
F1 macro averaged	68.8	59.6	68.7	65.6	66.3
Precision					
Normal/mild	99.2	93.0	97.4	97.0	96.7
Moderate	33.8	40.0	44.6	43.8	41.9
Severe	58.6	68.9	64.9	60.4	64.2
Weighted mean precision	92.7	88.1	92.1	91.5	91.3
Sensitivity					
Normal/mild	88.4	98.2	96.3	96.1	96.4
Moderate	71.3	20.4	53.7	55.6	48.1
Severe	78.5	47.7	56.9	44.6	52.3
Weighted mean sensitivity	86.8	90.3	91.5	90.9	90.9

*Note:* Higher values indicate better performance in percentages.

The fine‐tuned SGN achieved 99.2% precision for normal/mild cases, indicating high confidence for non‐stenotic conditions. The precision for moderate stenosis was low in all models, with the fine‐tuned SGN at 33.8% and DenseNet outperforming at 44.6%, indicating a high rate of false positive predictions. For severe stenosis, the original SpineNetV2 had 68.9% precision, while the fine‐tuned SGN achieved 58.6%.

### 3.3 Uncertainty Estimation for LSS Classification Model

The ECEs for all stenosis classes were around 0.15, confirming the poor calibration of the model's probabilities. The uncertainty quantification analysis of our spinal classification network (SGN) using MC dropout and TTA revealed distinct patterns in model confidence across severity grades of CCS (Figure S3). MC dropout demonstrated elevated uncertainty in clinically critical categories, with predictive variance values of 0.7 for moderate and severe stenosis, compared to 0.3 for normal/mild cases. In contrast, TTA exhibited its highest uncertainty (4.5 variance) in normal/mild classifications, while showing greater confidence in severe stenosis predictions (2.6 variance) and moderate cases (2.2 variance).

**FIGURE 3:**
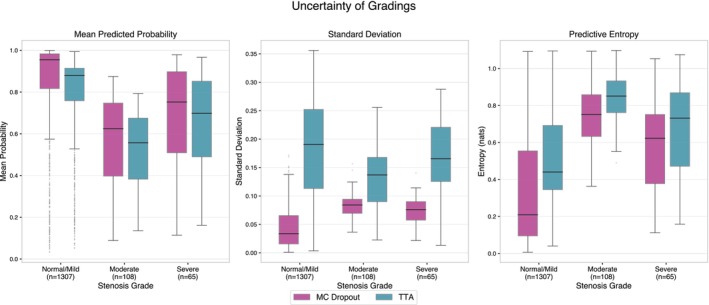
Box plots of uncertainty quantification metrics across Central Canal Stenosis (CCS) severity grades (Normal/Mild, *n* = 1307; Moderate, *n* = 108; Severe, *n* = 65) for MC Dropout (25 stochastic inference passes) and Test‐Time Augmentation (TTA, 9 augmented inference passes). Left: Mean predicted probability for the assigned severity class, reflecting model confidence. Centre: Standard deviation of the predicted class probability across stochastic iterations, quantifying prediction variability. Right: Predictive entropy, computed from the mean softmax distribution across stochastic forward passes, providing a complementary measure of overall prediction uncertainty. Across methods, entropy was lowest for Normal/Mild and higher for Moderate and Severe stenosis, consistent with greater diagnostic ambiguity in these classes.

**FIGURE 4:**
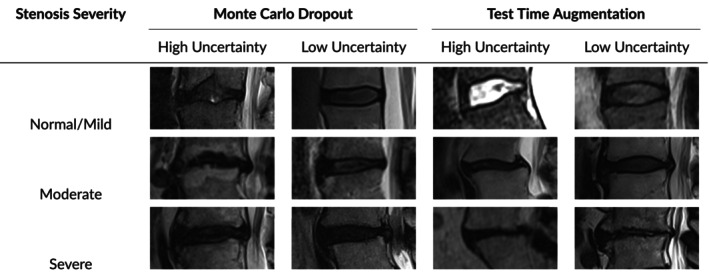
Visualization of high and low uncertainty examples across stenosis severity classes for Monte Carlo (MC) Dropout and Test Time Augmentation (TTA) methods.

Figure 3 illustrates the distribution of predicted probabilities and associated uncertainty (quantified by the standard deviation of the predicted probability for the true class) in the three classification classes for MC dropout and test time augmentation (TTA). For Normal/Mild cases, both methods produced high mean predicted probabilities of 0.97 and 0.88 for MC and TTA respectively, suggesting confident predictions. However, MC dropout exhibited a markedly lower spread (standard deviation) compared to TTA, indicating more consistent and certain predictions for this class. The median standard deviation was 0.031 for MC and 0.19 for TTA. For moderate cases, the mean predicted probabilities were lower overall (0.62 for MC and 0.57 for TTA), suggesting the model was less confident in assigning this label. Interestingly, the standard deviations were relatively low, especially for the MC dropout with a median of 0.088, suggesting that, although the model was not confident in its classification (lower mean), it was consistent (low uncertainty). For severe cases, both methods achieved higher mean predicted probabilities of 0.78 and 0.75 for MC and TTA respectively, but TTA again demonstrated greater variability. MC dropout maintained lower standard deviations (mean 0.075), implying more stable predictions. Overall, TTA consistently exhibited greater predictive variation (higher standard deviation) across all classes, suggesting that MC dropout provides more stable uncertainty estimates, particularly for Normal/Mild and Severe grades. For MC dropout, the thresholds above which a case was considered highly uncertain were 0.09, 0.06, and 0.09 for Normal/Mild, Moderate, and Severe cases, respectively. For TTA, the corresponding thresholds were 0.16, 0.25, and 0.22. The cases were marked as low uncertainty if the standard deviation of the predicted probabilities for the true class was below the following thresholds: 0.06, 0.01, and 0.05 for MC dropout, and 0.09, 0.11, and 0.12 for TTA, corresponding to the Normal/Mild, Moderate, and Severe categories, respectively. Figure 3 additionally presents predictive entropy as a complementary uncertainty measure; additional details on entropy‐based analyses and Expected Calibration Error (ECE) curves are provided in the Supporting Information (Sections S4 and S5). Misclassified cases, predominantly in the Normal/Mild and Severe categories, exhibited markedly higher predictive entropy than correctly classified cases (Figure S4). To further illustrate these findings, the representative IVD magnetic resonance volumes corresponding to cases with high and low predictive uncertainty for both MC dropout and TTA are provided in Figure 4.

These examples highlight that images associated with higher model uncertainty often reflect more degenerated cases or ambiguous anatomical features, emphasizing the practical value of uncertainty quantification for clinical decision support.

#### 3.3.1 Uncertainty‐Aware Consensus Performance in Spinal Stenosis Grading

**FIGURE 5:**
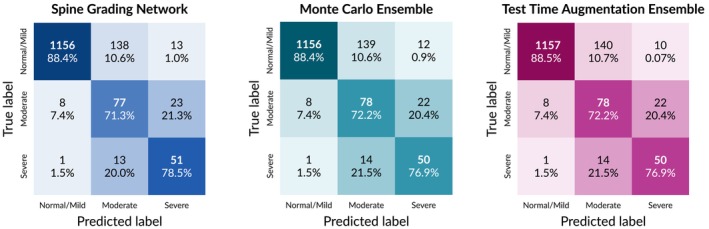
Results for fine‐tuned SpineNet Grading Network (SGN) on the test set. Confusion matrix, depicting the number of IVVs and absolute percentages for each class.

The confusion matrix of uncertainty quantification methods using majority voting is detailed in Figure 5. The application of majority voting to the dropout of MC improved the macro F1 score from 68.8% to 69.8%, indicating enhanced classification consistency, although the balanced accuracy decreased slightly to 77.4% compared to the baseline SGN performance of 79.4%. In contrast, the majority vote applied to TTA did not produce performance improvements, with a macro F1 score of 62.7% and a balanced accuracy remaining at 77.4%. These results suggest that the predictions of the consensus of MC dropout may increase the performance in the classification of CCS, while the consensus of the TTA does not significantly affect overall performance.

## 4 Discussion

This study demonstrates the viability of DL‐based automated CCS classification while rigorously addressing critical reliability concerns through uncertainty quantification. Our analysis of five architectures revealed that SGN fine‐tuning in the LumbarDISC data set [26] significantly improved diagnostic performance, achieving a balanced accuracy of 79.4%.

The pretrained SpineNetV2 architecture demonstrated strong performance in identifying normal/mild stenosis cases (98.2% accuracy), but exhibited concerning limitations in more serious conditions, achieving only 20.4% accuracy for moderate and 47.7% for severe stenosis (Table 1). This disparity resulted in a clinically inadequate balanced accuracy of 55.4%, despite the use of class balancing techniques during training [11]. We hypothesize that this reflects a fundamental challenge of generalization in medical AI, where models trained on a patient population (Genodisc dataset) may struggle when applied to new clinical populations (LumbarDISC dataset) due to differences in imaging protocols, stenosis prevalence, or diagnostic criteria. Incorporating uncertainty estimation could help address this generalizability challenge by providing not only a prediction but also a confidence measure, allowing difficult or out‐of‐distribution cases to be flagged for further human review.

In addition to SGN, we benchmarked five different model architectures. DenseNet achieved the best performance (69% balanced accuracy), although still significantly below the fine‐tuned SGN. Despite the overall good performance of SGN, the confusion matrix revealed persistent challenges in distinguishing moderate from severe cases (21.3% misclassification rate), reflecting known diagnostic difficulties even among human experts [2]. The class imbalance emerged as a critical factor that influences both model performance and uncertainty patterns. After assessing model’s poor calibration using ECE, we applied two well‐known methods to estimate uncertainty, namely MC dropout and TTA.

The differences in uncertainty quantification between MC dropout and TTA could be useful in real clinical work‐flows. MC dropout showed lower uncertainty across all classes, especially for *normal/mild* and *severe* cases. This suggests that its predictions are more stable and could be trusted more when the model is confident. In practice, this means that MC dropout might be helpful to automatically handle mild and severe cases, saving radiologists time by reducing the number of images that need manual review. However, TTA showed higher uncertainty, especially in the *normal/mild* cases. This could actually help spot unusual or borderline normal cases that still deserve a second revision. For example, a case that looks mild but has high uncertainty might reflect an early‐stage or a dynamic issue that is not obvious, and this extra flag could prompt additional imaging or follow‐up.

For moderate cases—which are often the hardest to classify—both methods resulted in lower confidence, but the uncertainty was still relatively low, especially for MC dropout. This pattern suggests that the model is not confident about the label (low probability), but consistent in that uncertainty. In a clinical setting, these cases could be automatically flagged for further review, helping clinicians focusing on the more challenging diagnoses. In summary, MC dropout could be used to automate decisions on clearer cases, while TTA might be better for identifying subtle or underconfident presentations that need more attention. Using both methods together could support a more balanced and reliable screening process, helping to prioritize the right cases for follow‐up or expert review. A minor point worth noting—and a potential limitation—is that we used 25 iterations for MC dropout but only 9 iterations for TTA. This means the uncertainty estimates from MC dropout may be more reliable simply because they are based on more iterations. Furthermore, MC dropout tends to have a smaller effect on the model predictions, since randomly dropping neurons during inference does not drastically alter the output of a well‐trained model. In contrast, test‐time augmentation can introduce more significant changes, as the input image itself is modified, which can lead to greater variation in predictions. This pattern aligns with the class performance metrics reported in Figure 3, where MC dropout achieved its highest class accuracy for normal/mild cases and relatively lower accuracy for moderate and severe stenosis.

The implementation of uncertainty‐aware consensus methods yielded method‐dependent improvements. MC dropout with majority voting slightly improved diagnostic consistency in terms of F1 score while maintaining balanced accuracy (77.4% vs. baseline 79.4%), suggesting clinical viability for multiclass grading. The TTA consensus did not show performance gains, probably due to the inherent variability in the data in the presentation of early stage stenosis that simple augmentation strategies cannot resolve. This suggests that, under TTA, the model is less certain when predicting normal/mild cases compared to more severe categories. In particular, TTA achieved its highest‐class accuracy for severe stenosis, MC dropout's consensus predictions improve reliability for multiclass stenosis grading, even with a slight drop in the accuracy. From a deployment perspective, the additional computational cost of uncertainty estimation remains compatible with clinical use. Although stochastic inference increases runtime relative to the base SpineNetV2 model, these absolute processing times remain affordable, and future implementations could further reduce latency through parallelized inference.

Although demonstrating improved reliability in CCS classification, our multicenter retrospective design requires validation across various clinical populations and imaging protocols. In fact, a critical limitation of this study lies in the evaluation of uncertainty estimation exclusively on in‐distribution data. The performance and reliability of the model under distribution shift scenarios, common in real‐world clinical deployments, remain uncharacterized. Future studies should validate these uncertainty quantification methods on external datasets that exhibit natural variations in imaging protocols and patient demographics. Future work should examine whether predictive uncertainty is consistently higher in misclassified cases, further supporting the clinical utility of uncertainty‐based case flagging. Based on previous evidence that demonstrated the superior robustness of deep ensembles to distribution shifts [35], future research should investigate ensemble‐based uncertainty frameworks for the prediction of spinal stenosis in heterogeneous clinical environments. Furthermore, to address the higher model confidence observed in majority classes, future studies may explore training strategies to mitigate the effects of class imbalance.

Additionally, to improve clinical trustworthiness, the implementation of post hoc calibration techniques such as temperature scaling [25] may be beneficial. These methods align predicted confidence with actual diagnostic frequencies, essential for assessing difficult cases based on uncertainty thresholds for review by clinicians. Based on our findings, to bridge the gap between AI and clinical application, we could propose a potential clinical workflow in which MC dropout serves as a screening tool to prioritize cases with a high probability of pathology, while cases with high predictive uncertainty are flagged for a more in‐depth review by clinicians.

## Author Contributions

**Adriana Brenzikofer:** conceptualization, methodology, investigation, software, data curation, writing – original draft, writing – review and editing, visualization. **Maria Monzon:** conceptualization, methodology, validation, writing – original draft, writing – review and editing, supervision, software, investigation, project administration, visualization. **Fabio Galbusera:** writing – review and editing, methodology, supervision. **Zina‐Mary Manjaly:** writing – review and editing, funding acquisition. **Andrea Cina:** conceptualization, writing – original draft, writing – review and editing, supervision, project administration, methodology, investigation, validation. **Catherine R. Jutzeler:** writing – review and editing, funding acquisition, resources, supervision, validation.

## Funding

Strategic Focus Area “Personalized Health and Related Technologies (PHRT),” ETH Domain (Swiss Federal Institute of Technology), Grant Number: 380; Ambizione Grant. Grant number: #PZ00P3_186101, CRJ and the Schulthess Klinik Research Fund (FG).

## Conflicts of Interest

C.R.J. serves as a scientific consultant for Abbvie and Mitsubishi Takeda; however, this role did not influence the design, conduct, or reporting of this study. The other authors declare no conflicts of interest.

## Supporting information


**Figure S1:** Confusion matrices for different deep learning architectures on lumbar central canal stenosis classification. Results show model classified samples across *Normal/Mild, Moderate*, and *Severe* stenosis classes.
**Figure S2:** Visualization of image augmentation transformations applied for Test Time Augmentation (TTA)
**Figure S3:** Box plots show mean predicted probabilities (top row) and standard deviations for predicted severity class (Normal/Mild, Moderate, Severe) using Monte Carlo dropout (MC) and Test Time Augmentation (TTA).
**Figure S4:** Predictive entropy of CCS grading predictions, stratified by classification outcome, for MC Dropout (*T* = 25 passes) and Test‐Time Augmentation (TTA, *T* = 9 passes). For each spine level, repeated stochastic predictions generated class‐probability vectors which were averaged to obtain the final predictive distribution. Higher entropy indicates greater uncertainty in the final stenosis grade, whereas lower entropy indicates a more focused and confident class assignment. Boxes show the distribution of for correctly classified and misclassified spine levels within each stenosis grade. Brackets indicate statistically significant differences between correct and incorrect predictions (one‐sided Mann–Whitney *U* test, ***p < 0.001). Misclassified Normal/Mild and Severe cases showed significantly higher entropy under both methods, whereas no significant difference was observed for Moderate stenosis, consistent with the greater ambiguity of this intermediate class.
**Figure S5:** Reliability curves and Expected Calibration Error (ECE) for Monte Carlo dropout (MC) and Test Time Augmentation (TTA). Lower ECE indicates better agreement between predicted confidence and empirical accuracy.
**Table S1:** Hyperparameters of the model architectures with the best performance according to the balanced accuracy.

## Data Availability

The data that support the findings of this study are openly available in RSNA‐LumbarDISC at https://doi.org/10.1148/dataset.lsdd.2025.
