# Supplementary material for: Uncertainty Quantification of Central Canal Stenosis Deep Learning Classifier From Lumbar Sagittal T2‐Weighted MRI
Source: JOR Spine. 2026 Jun 8;9(2):e70184. doi: 10.1002/jsp2.70184 (PMC13246319; doi:10.1002/jsp2.70184)
Supplement: Supplementary file 1 — Figure S1: Confusion matrices for different deep learning architectures on lumbar central canal stenosis classification. Results show model classified samples across Normal/Mild, Moderate, and Severe stenosis classes. Figure S2: Visualization of image augmentation transformations applied for Test Time Augmentation (TTA) Figure S3: Box plots show mean predicted probabilities (top row) and standard deviations for predicted severity class (Normal/Mild, Moderate, Severe) using Monte Carlo dropout (MC) and Test Time Augmentation (TTA). Figure S4: Predictive entropy of CCS grading predictions, stratified by classification outcome, for MC Dropout (T = 25 passes) and Test‐Time Augmentation (TTA, T = 9 passes). For each spine level, repeated stochastic predictions generated class‐probability vectors which were averaged to obtain the final predictive distribution. Higher entropy indicates greater uncertainty in the final stenosis grade, whereas lower entropy indicates a more focused and confident class assignment. Boxes show the distribution of for correctly classified and misclassified spine levels within each stenosis grade. Brackets indicate statistically significant differences between correct and incorrect predictions (one‐sided Mann–Whitney U test, ***p < 0.001). Misclassified Normal/Mild and Severe cases showed significantly higher entropy under both methods, whereas no significant difference was observed for Moderate stenosis, consistent with the greater ambiguity of this intermediate class. Figure S5: Reliability curves and Expected Calibration Error (ECE) for Monte Carlo dropout (MC) and Test Time Augmentation (TTA). Lower ECE indicates better agreement between predicted confidence and empirical accuracy. Table S1: Hyperparameters of the model architectures with the best performance according to the balanced accuracy. [file JSP2-9-e70184-s001.pdf]

Supporting Information

This appendix provides a comprehensive overview of the CCS grading using the deep learning models hyperparameter optimization process, benchmarking strategy, and detailed classification performance analysis.

| CCS classification model and hyperparameter optimization and benchmarking

Building reliable deep learning models for CCS classification involves systematic evaluation across various architectures and optimizing training parameters. With significant class imbalance in stenosis datasets (88% normal/mild, 7% moderate, 5% severe), our strategy focuses on balanced accuracy to ensure consistent performance across all severity levels.

To optimize the performance of the models, we conducted a comprehensive hyperparameter random search, testing different batch sizes (16, 32) and learning rates (10^{-3} to 10^{-6}) with variable dropout rates (0-0.9 in 0.1 steps). Models were trained for 30-130 epochs with early stopping after 20 epochs if there was no improvement in validation loss. Warm-up and cool-down mechanisms were used to stabilize training and enhance convergence. The warm-up was linear over 1000 batches with a factor of 0.01. Cooldown reduced the learning rate by 0.5 on a plateau if validation loss did not improve after 5 epochs, using a minimum learning rate of 10^{-7}. To improve training and model generalization, two data augmentations were applied with a probability of 0.5, allowing multiple transformations per sample: random zooming from 90 to 110%, maintaining IVD volume size, and random Gaussian noise with a mean of 0 and a standard deviation of 0.01. We selected the optimal set of hyperparameters based on the balanced accuracy performance in the validation data set.

The hyperparameter optimization process was carried out through a systematic random search to maximize the balanced accuracy at all stenosis severity levels. Although DenseNet employed a weighted random sampler strategy, all other models utilized weighted cross-entropy to address class imbalance. Table S1 presents the final hyperparameter configuration for each model architecture evaluated in this study.

TABLE S1 Hyperparameters of the model architectures with the best performance according to the balanced accuracy

Parameters	fine-tuned SGN	DenseNet	ResNet	EfficientNet
Batch size	16	16	16	16
Learning rate	10^{-6}	10^{-3}	10^{-4}	10^{-4}
Weighted random sampler	False	True	False	False
Weighted Cross Entropy	True	False	True	True
Dropout rate	0.8	0.2	0.2	0.3

Five deep learning architectures were benchmarked for their ability to classify CCS severity. Figure S1 shows confusion matrices that highlight key insights into each model's classification behavior. The confusion matrices reveal consistent interclass confusion between moderate and severe stenosis in all architectures, with misclassification rates ranging from 17.6% to 23.1%.

DenseNet, ResNet, and EfficientNet demonstrated moderate performance, while original SpineNetV2 showed limited generalisability to the target dataset. DenseNet achieved the second highest performance with a balanced

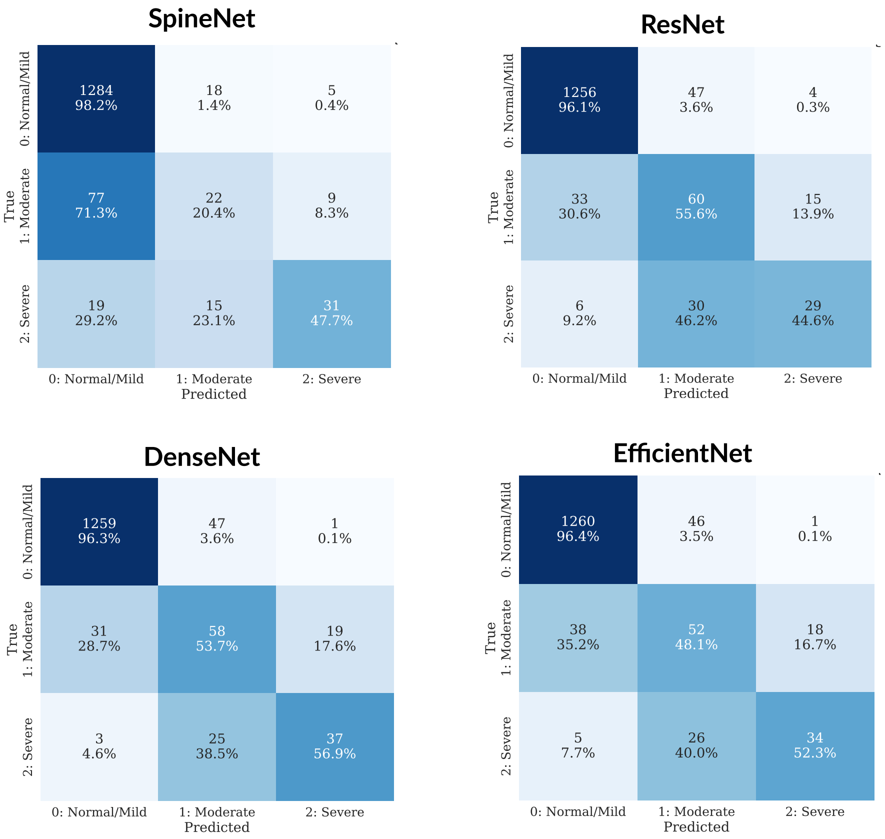

FIGURE S1 Confusion matrices for different deep learning architectures on lumbar central canal stenosis classification. Results show model classified samples across *Normal/Mild*, *Moderate* , and *Severe* stenosis classes.

accuracy of 69. 0% and a competitive macro F1 score of 68.7%. ResNet and EfficientNet exhibited moderate performance levels, with both models achieving similar balanced accuracy (65.4% and 65.6%, respectively) and macro F1 scores (65.6% and 66.3%, respectively). In contrast, the original SpineNet architecture demonstrated a significant performance limitation, achieving high accuracy (98.2%) for the majority normal/mild stenosis class but showing poor performance for minority classes, with only 20.4% accuracy for moderate stenosis and 47.7% for severe stenosis, resulting in an overall balanced accuracy of just 55.4%.

Test Time Augmentations

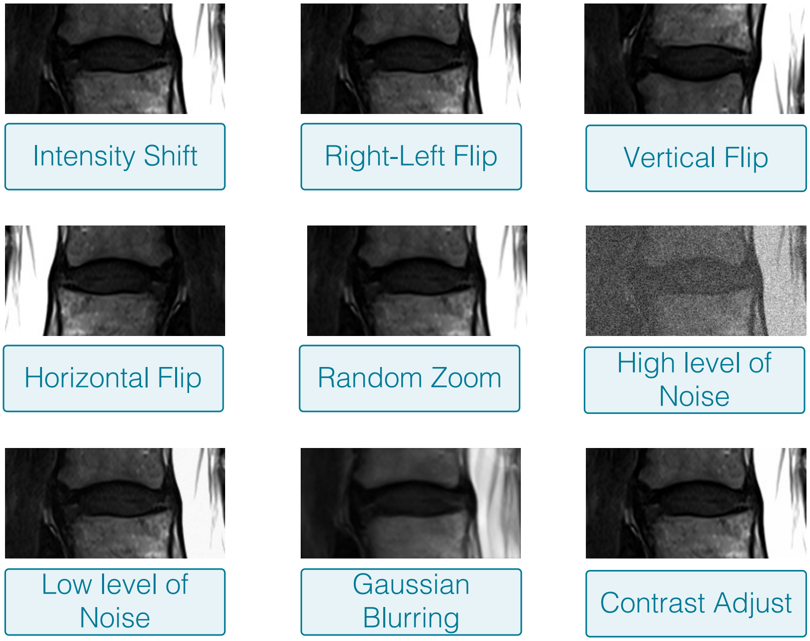

FIGURE S2 Visualization of image augmentation transformations applied for Test Time Augmentation (TTA)

The implementation of TTA used nine image perturbations the model's sensitivity to controlled input variation during inference, rather than to simulate anatomically realistic MRI acquisitions. Figure S2 illustrates the nine image perturbations used during TTA inference.

- Intensity shift: random intensity offset in the range [-0.05, 0.05] to perturb global signal levels
- Contrast Adjustment: gamma correction with values between 0.95 and 1.05 to perturb image contrast
- Gaussian Noise Addition (low-level): noise perturbation with standard deviation of 0.01 to introduce a mild noise perturbation
- Gaussian Noise Addition (high-level): noise perturbation with standard deviation of 0.2 to introduce a strong noise perturbation
- Right-left flip: image reflection used as a geometric perturbation
- Vertical Flip: image reflection used as a geometric perturbation
- Horizontal Flip: image reflection used as a geometric perturbation
- Random Zoom: scale variation between 90% and 110% while preserving the final intervertebral disc volume dimensions
- Gaussian Blurring: smoothing perturbation to reduce local high-frequency detail

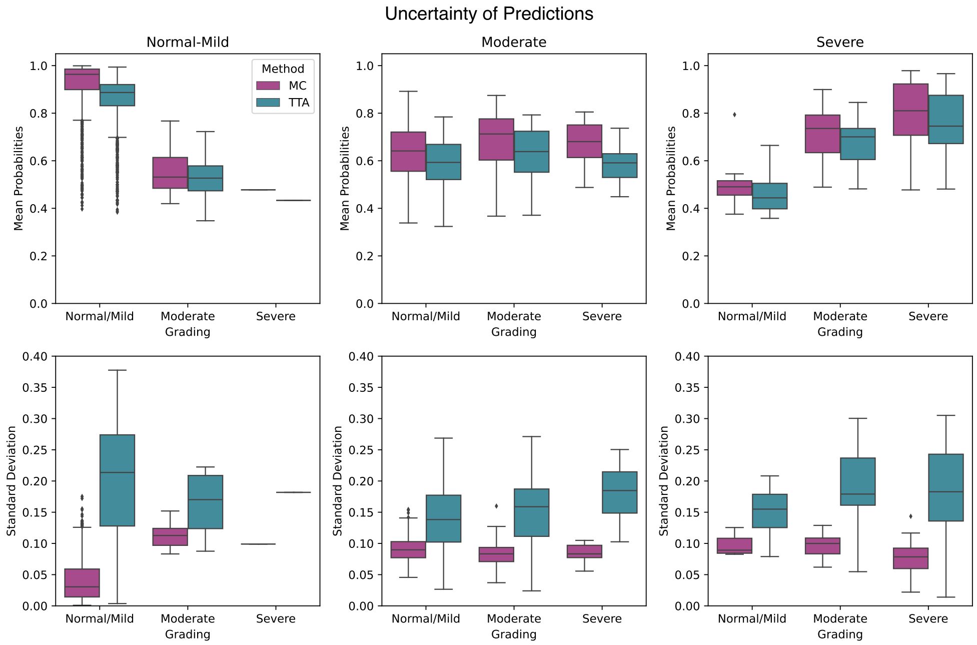

FIGURE S3 Box plots show mean predicted probabilities (top row) and standard deviations for predicted severity class (Normal/Mild, Moderate, Severe) using Monte Carlo dropout (MC) and Test Time Augmentation (TTA).

Uncertainty Estimation

A comprehensive comparative visualization of uncertainty estimates between Monte Carlo (MC) dropout and Test Time Augmentation (TTA) methods across all stenosis severity classes is presented in Figure S3. The stratified approach assesses each uncertainty quantification method when the true diagnosis is known.

Figure S3 shows uncertainty patterns for predicted classes, with results organized by true stenosis severity, offering insights into model confidence and reliability across categories. MC dropout maintains high confidence across all classes, with median probabilities of 0.963 for Normal/Mild, 0.673 for Moderate, and 0.762 for Severe stenosis, indicating strong epistemic uncertainty quantification. Uncertainty, measured by standard deviations, increases with stenosis severity: Normal/Mild (median 0.031, IQR 0.014-0.060), Moderate (median 0.087, IQR 0.074-0.099), Severe (median 0.088, IQR 0.070-0.104). This trend matches clinical expectations, as complex pathologies introduce more ambiguity. TTA shows lower confidence with median probabilities of 0.887 for Normal/Mild, 0.605 for Moderate, and 0.701 for Severe stenosis, reflecting sensitivity to data variation and capturing aleatoric uncertainty. Uncertainty is highest in Normal/Mild cases (median 0.213, IQR 0.128-0.273), followed by Severe (median 0.179, IQR 0.134-0.233), and lowest in Moderate (median 0.148, IQR 0.108-0.185). This suggests TTA is sensitive to early pathology variations and severe distortions. MC dropout has narrow uncertainty distributions, most spread in Moderate and Severe cases (IQR 0.025 and 0.034), while TTA's broader distributions, especially in Normal/Mild cases (IQR 0.145), show more sensitivity to input changes.

| Predictive entropy and classification errors

To complement the standard deviation-based analysis, we evaluated predictive entropy across repeated model predictions. For each intervertebral volume, MC Dropout and TTA generated multiple class-probability estimates, which were averaged to obtain a final probability profile for each case. Predictive entropy was then calculated from this average distribution. For each input sample, the model produced T softmax probability vectors, where $T = 25$ for MC Dropout and $T = 9$ for TTA. Let $p^{(t) \in \mathbb{R}^K}$ denote the softmax output at stochastic pass t, with $K = 3$ classes. The mean predictive distribution was then computed as

$$\bar{p} = \frac{1}{T} \sum_{t=1}^{T} p^{(t)}.$$

Predictive entropy was calculated from this averaged distribution as

$$H[\bar{p}] = -\sum_{k=1}^{K} \bar{p}_k \log \bar{p}_k.$$

This quantity summarizes the total uncertainty of the predictive distribution and complements the standard deviation of the predicted class probability used in the main analysis. Predictive entropy reflects how clearly the model favours one stenosis category over the others. Lower entropy indicates a more concentrated prediction, whereas higher entropy reflects greater ambiguity in the final class assignment. As shown in Supplementary Figure S4, misclassified Normal/Mild and Severe cases had significantly higher entropy than correctly classified cases for both MC Dropout and TTA. No significant difference was observed for Moderate stenosis, consistent with the greater ambiguity of this intermediate class and the overlap between adjacent grades. Overall, these findings indicate that higher predictive entropy is associated with a higher likelihood of classification error in the more clearly separable classes.

| Calibration analysis

Calibration was further assessed using Expected Calibration Error (ECE). Figure S5 shows that MC dropout was better calibrated overall than TTA, although both methods displayed residual class-specific miscalibration. These findings further support the use of explicit uncertainty estimates in addition to raw predicted probabilities.

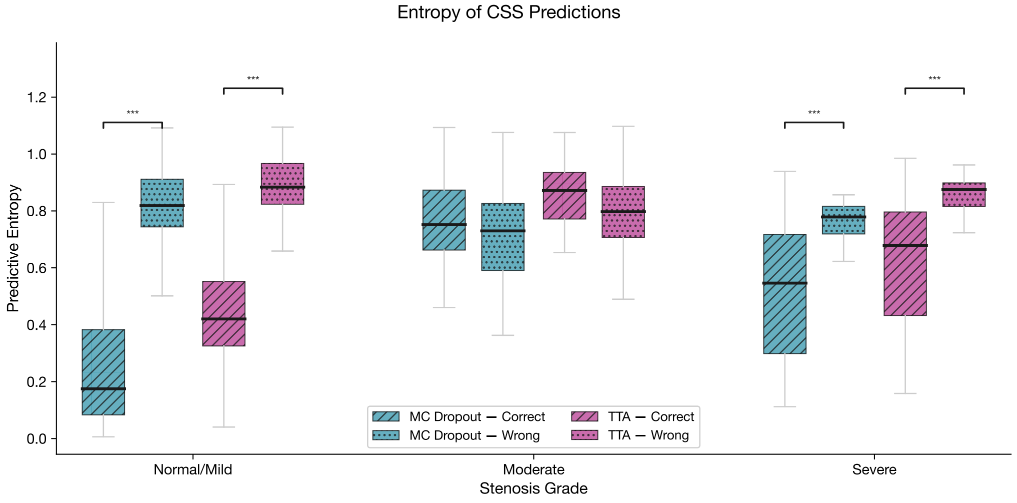

FIGURE S4 Predictive entropy of CCS grading predictions, stratified by classification outcome, for MC Dropout (T=25 passes) and Test-Time Augmentation (TTA, T=9 passes). For each spine level, repeated stochastic predictions generated class-probability vectors which were averaged to obtain the final predictive distribution. Higher entropy indicates greater uncertainty in the final stenosis grade, whereas lower entropy indicates a more focused and confident class assignment. Boxes show the distribution of for correctly classified and misclassified spine levels within each stenosis grade. Brackets indicate statistically significant differences between correct and incorrect predictions (one-sided Mann–Whitney U test, $^{***}p < 0.001$). Misclassified Normal/Mild and Severe cases showed significantly higher entropy under both methods, whereas no significant difference was observed for Moderate stenosis, consistent with the greater ambiguity of this intermediate class.

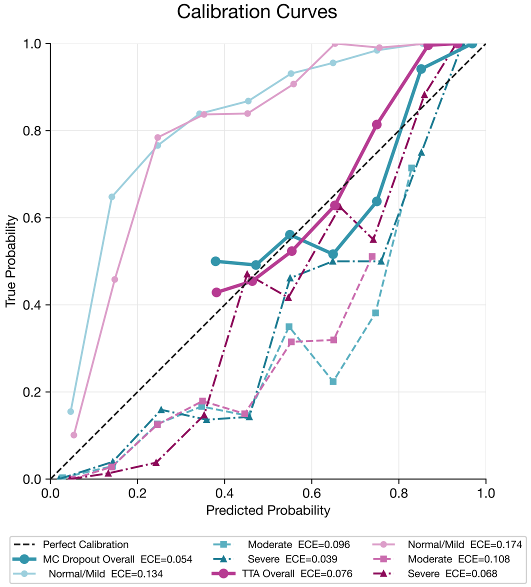

FIGURE S5 Reliability curves and Expected Calibration Error (ECE) for Monte Carlo dropout (MC) and Test Time Augmentation (TTA). Lower ECE indicates better agreement between predicted confidence and empirical accuracy.
